# The use of non-steroid anti-inflammatory drugs during radical resection correlated with the outcome in non-small cell lung cancer

**DOI:** 10.1186/s12957-023-03247-8

**Published:** 2023-11-21

**Authors:** Renzhong Cai, Xuqiang Liao, Gao Li, Jia Xiang, Qianwen Ye, Minbiao Chen, Shouhan Feng

**Affiliations:** 1 Department of Thoracic Surgery, Hainan General Hospital, Hainan Affiliated Hospital of Hainan Medical University, Haikou City, Hainan Province 570311 People’s Republic of China; 2 Department of Oncology, Hainan Hospital of PLA General Hospital, Sanya City, Hainan Province 572000 People’s Republic of China; 3 Department of Oncology, Huzhou Traditional Chinese Medicine Hospital Affiliated to Zhejiang Chinese Medical University, Huzhou City, Zhejiang Province 313000 People’s Republic of China

**Keywords:** Lung cancer, Non-steroid anti-inflammatory drugs, Surgery, Disease-free survival, Overall survival

## Abstract

**Aims:**

The use of non-steroid anti-inflammatory drugs (NSAIDs) is conventional in management of postoperative pain in cancer patients, and further investigations have reported that some of these drugs correlated with the outcome in cancers. However, the prognostic value of the use of NSAIDs during surgery in non-small cell lung cancer (NSCLC) patients has been less addressed.

**Methods:**

NSCLC patients staged I–III are retrospectively enrolled, and the data of the use of NSAIDs during surgery are collected. Patients are divided into two subgroups according to the use intensity (UI) (low or high) of the NSAIDs, which was calculated by the accumulate dosage of all the NSAIDs divided by the length of hospitalization. The differences of the clinical features among these groups were checked. And the disease-free survival (DFS) and overall survival (OS) differences in these groups were compared by Kaplan–Meier analysis; risk factors for survival were validated by using a Cox proportional hazards model.

**Results:**

The UI was significant in predicting the DFS (*AUC* = 0.65, 95% *CI*: 0.57–0.73, *P* = 0.001) and OS (*AUC* = 0.70, 95% *CI*: 0.59–0.81, *P* = 0.001). Clinical features including type of resection (*P* = 0.001), N stages (*P* < 0.001), and TNM stages (*P* = 0.004) were significantly different in UI low (< 74.55 mg/day) or high (≥ 74.55 mg/day) subgroups. Patients in UI-high subgroups displayed significant superior DFS (log rank = 11.46, *P* = 0.001) and OS (log rank = 7.63, *P* = 0.006) than the UI-low ones. At last, the UI was found to be an independent risk factor for DFS (*HR*: 0.52, 95% *CI*: 0.28–0.95, *P* = 0.034).

**Conclusions:**

The use of NSAIDs during radical resection in NSCLC patients correlated with the outcome and patients with a relative high UI has better outcome.

## Introduction

Lung cancer is still a great health threat worldwide [1] with non-small cell lung cancer (NSCLC) which accounts as the predominant pattern [2]. In recent years, with the success of neoadjuvant, adjuvant immunotherapies [3, 4], and target therapies [5, 6], the overall survival (OS) of NSCLC patients was greatly improved even in those locally advanced cases. Nonetheless, radical resection was still the most important treatment for the majority of the patients; however, some complications like pain are still a serious problem to harm the quality of life for the patients after surgery.

It was reported the prevalence of clinically relevant postoperative pain for lung cancer patients could be up to 63% for those received thoracotomy [7] and could persist to 36 months (m) in 17.4% patients [8]. Clinically, opioids and non-steroid anti-inflammatory drugs (NSAIDs) are the most used agents to alleviate the postoperative pain in these patients [9], the latter of which include those unselected COX inhibitors like aspirin, diclofenac, and ibuprofen and those selected COX-2 inhibitors like rofecoxib and celecoxib [10]. Interestingly, a great number of previous studies have indicated some of these NSAIDs may have additional role in regulating lung cancer cells besides its function in anti-inflammation and alleviate pain. For example, aspirin could improve cisplatin resistance by inhibiting cancer cell stemness [11] or reduce the metastasis of the cancer cells to regional lymph nodes [12]; ibuprofen could enhance the effect of cisplatin by suppressing the heat shock protein 70 in cancer cells [13], which indicated a positive role of these agents in anti-cancer. On the contrary, celecoxib could induce the epithelial-mesenchymal transition and increase the risks of cancer metastasis [14]. Based on these facts, some clinical observations have conducted to explore the role of the NSAIDs in lung cancer patients as it was found that the combined use of aspirin with osimertinib [15] or immunotherapies [16] contributes to the good survival in patients; however, no such role was detected for the combination with celecoxib [17, 18]. Of note, all these trials were performed in advanced or metastatic settings. Up to date, only one study has explored the use of the NSAIDs in postoperative stages I–III NSCLC patients [19], but it only includes the use of indomethacin and ibuprofen which aimed to alleviate the postoperative fever; other types of NSAIDs were not included. Nonetheless, none of the study has explored the prognostic value of the use of NSAIDs during surgery in these patients.

In this study, we sought to explore the prognostic value of the use of NSAIDs during radical resection in stages I–III NSCLC.

## Methods

### Data collection

NSCLC patients who experienced radical resection in Hainan Hospital of PLA General Hospital from December 2012 to May 2020 were retrospectively enrolled. Clinic data including age, sex, type of resection, pathology, smoking or alcohol status, and comorbidity (hypertension or diabetes mellitus) were collected. Patients met any of the following criteria which were ruled out: (1) receive any kind or duration of neoadjuvant therapies; (2) with suspected remote lesions by examinations before surgery; (3) with previous secondary malignant disease; (4) with a use history of any kind of NSAIDs due to cardiovascular or cerebrovascular disease and others, or those with oxycodone/acetaminophen; and (5) follow up problems (refuse or lost). The study followed the principles stated in the Declaration of Helsinki and was approved by the Ethics Committee of Hainan Hospital of PLA General Hospital (ID: S2023-12). Written informed consent was not required because of its retrospective nature.

### NSAIDs data collection and patient assignment

The categories of NSAIDs used in this study include aspirin/aspirin DL-lysine, loxoprofen, ibuprofen, diclofenac sodium, and ketorolac tromethamine, and no records of celecoxib were registered. The accumulated dosage (AD) of these agents is figured out individually according to the package insert and was summed up with a convert ratio at 1 to each other for the convenience of subsequent calculation. All the NSAIDs were used in these patients aimed to alleviate postoperative pain except five patients who experienced fever. Patients were then assigned into two subgroups based on the use intensity (UI), which was calculated by the sum of all the dosage of the NSAIDs (mg) divided by the length of hospitalization (days, d). In addition, patients were also divided into use status (US, no/yes) of which the ones with any kind and any dosage of NSAIDs prescription were assigned into the US yes groups.

### Set the study endpoints

The follow-up was started after the resection with an interval of 3–6 months in the first 2 years (y) and then annually. DFS and OS were selected as the primary endpoints for the study as described previously [20], and the last follow-up point was stopped in May 2023.

### Statistical analysis

The significance of UI, US, and AD in predicting DFS and OS was checked by the receiver operating characteristic curve (ROC) analysis, after comparing the area under the curve (AUC) of these indexes; patients were then assigned into UI low or high subgroups according to the optimal cut-off point (taken DFS as the endpoint). Clinical features in these subgroups were tested by chi-square test. The DFS and OS differences among UI low or high and US no or yes subgroups were analyzed by Kaplan–Meier analysis and subsequent log-rank tests. A Cox proportional hazards model was used to validate the risk factors for the outcome with the iterative forward LR method. Double side *P* < 0.050 was deemed as statistically significant. All the data were processed by using SPSS 20.0 (SPSS Inc., Chicago, IL, USA).

## Results

### General features of the patients

**Fig. 1:**
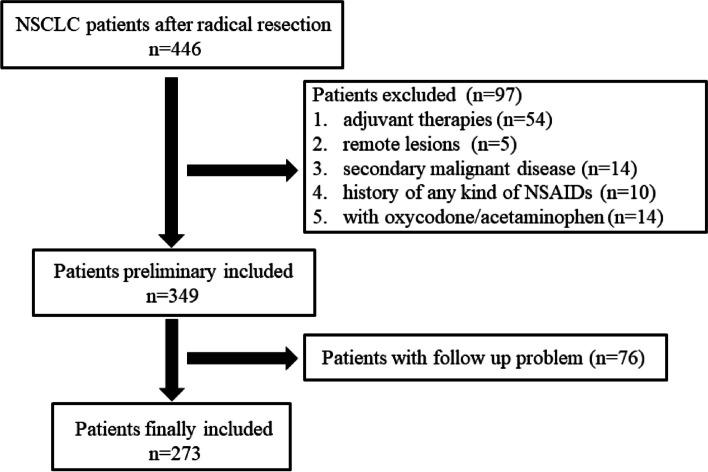
A flowchart of patient enrollment

A total of 273 patients were enrolled in the last analytic set (Fig. 1) with 136 males and 137 females, the median age of the patients was 58 years (range: 23–84 years), and the median follow-up was 52 months (range: 2–128 months). At the end of the follow-up, 10 patients in stage I, 7 patients in stage II, and 8 patients in stage III died. The median of the AD was 1260 mg (range: 0–8260 mg), and the median of the length of hospitalization was 17 days (range: 6–54 days). The distribution of the categories of NSAIDs was as follows: none (*n* = 77); single agents: aspirin/aspirin-dl-lysine (*n* = 4, 2 for each), loxoprofen (*n* = 57), and diclofenac sodium (*n* = 67); and two agents: loxoprofen + diclofenac sodium (*n* = 40), loxoprofen + ketorolac tromethamine (*n* = 1), loxoprofen + ibuprofen (*n* = 1), ibuprofen + diclofenac sodium (*n* = 2), aspirin + diclofenac sodium (*n* = 1), and ketorolac tromethamine + diclofenac sodium (*n* = 19), and the rest of the patients (*n* = 4) used 3 categories.

### UI, US, and AD in predicting the DFS and OS

**Fig. 2:**
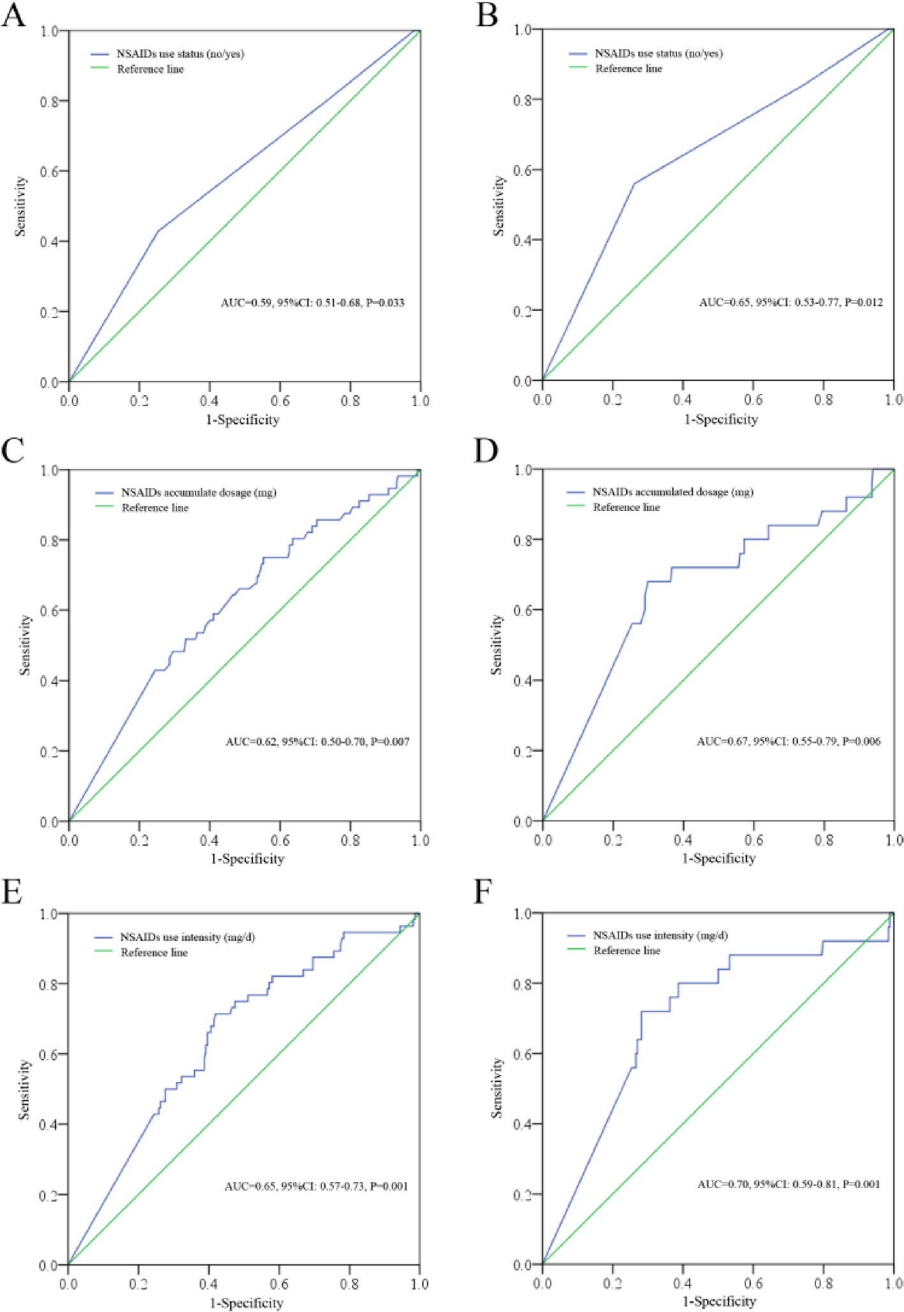
The significance of US, AD, and UI in predicting the survival. **A** US no or yes in predicting the DFS. **B** US no or yes in predicting the OS. **C** AD in predicting the DFS. **D** AD in predicting the OS. **E** UI in predicting the DFS. **D** UI in predicting the OS. US, use status; AD, accumulated dosage; UI, use intensity; DFS, disease-free survival; OS, overall survival

By ROC analysis, the UI was significant in predicting the DFS (*AUC* = 0.65, 95% *CI*: 0.57–0.73, *P* = 0.001) with a sensitivity and specificity at 71.40% and 58.10%, respectively, and OS (*AUC* = 0.70, 95% *CI*: 0.59–0.81, *P* = 0.001) with a sensitivity and specificity at 72.00% and 71.80%, respectively. In addition, the US was also significant in predicting DFS (*AUC* = 0.59, 95% *CI*: 0.51–0.68, *P* = 0.033) and OS (*AUC* = 0.65, 95% *CI*: 0.53–0.77, *P* = 0.012). We also checked the AD in predicting the survival, and the results suggested that it was also significant in predicting the DFS (*AUC* = 0.62, 95% *CI*: 0.50–0.70, *P* = 0.007) and OS (*AUC* = 0.67, 95% *CI*: 0.55–0.79, *P* = 0.006) (Fig. 2). The UI displayed the highest AUC among these indexes; we then divided the patients into UI low (< 74.55 mg/d, *n* = 131) or high (≥ 74.55 mg/d, *n* = 142) subgroups according to the optimal cut-off point for DFS. Further comparison for clinical features indicated that type of resection (*P* = 0.001), N stages (*P* < 0.001), and TNM stages (*P* = 0.004) were significantly different in UI low or high subgroups (Table 1).

**Table 1:** Differences of the parameters among UI low or high subgroups

	**NSAIDs UI**			
	**No. of the patients**	**Low**	**High**	***P***
**Age (y)**				0.463
< 60	157	72	85	
≥ 60	116	59	57	
**Sex**				0.070
Male	136	73	63	
Female	137	58	79	
**Type of resection**				0.001*
Lobectomy	208	112	96	
Segmentectomy	65	19	46	
**Pathology**				0.181
ADC	252	117	135	
SCC	14	10	4	
Others	7	4	3	
**Smoking status**				0.143
Never	195	88	107	
Current + former	78	43	35	
**Alcohol status**				0.608
Never	182	85	97	
Current + former	91	46	45	
**Comorbidity**				0.580
With	69	31	38	
Without	204	100	104	
**T stages**				0.686
T_1_ + T_2_	267	129	138	
T_3_ + T_4_	6	2	4	
**N stages**				< 0.001*
N_0_	241	106	135	
N_1_ + N_2_	32	25	7	
**TNM stages**				0.004*
I	237	105	132	
II	22	17	5	
III	14	9	5	

*UI* use intensity, *NSAIDs* non-steroid anti-inflammatory drugs, *ADC* adenocarcinoma, *SCC* squamous carcinoma

^*^With significant difference

### The survival differences in US yes or no and UI low or high subgroups

**Fig. 3:**
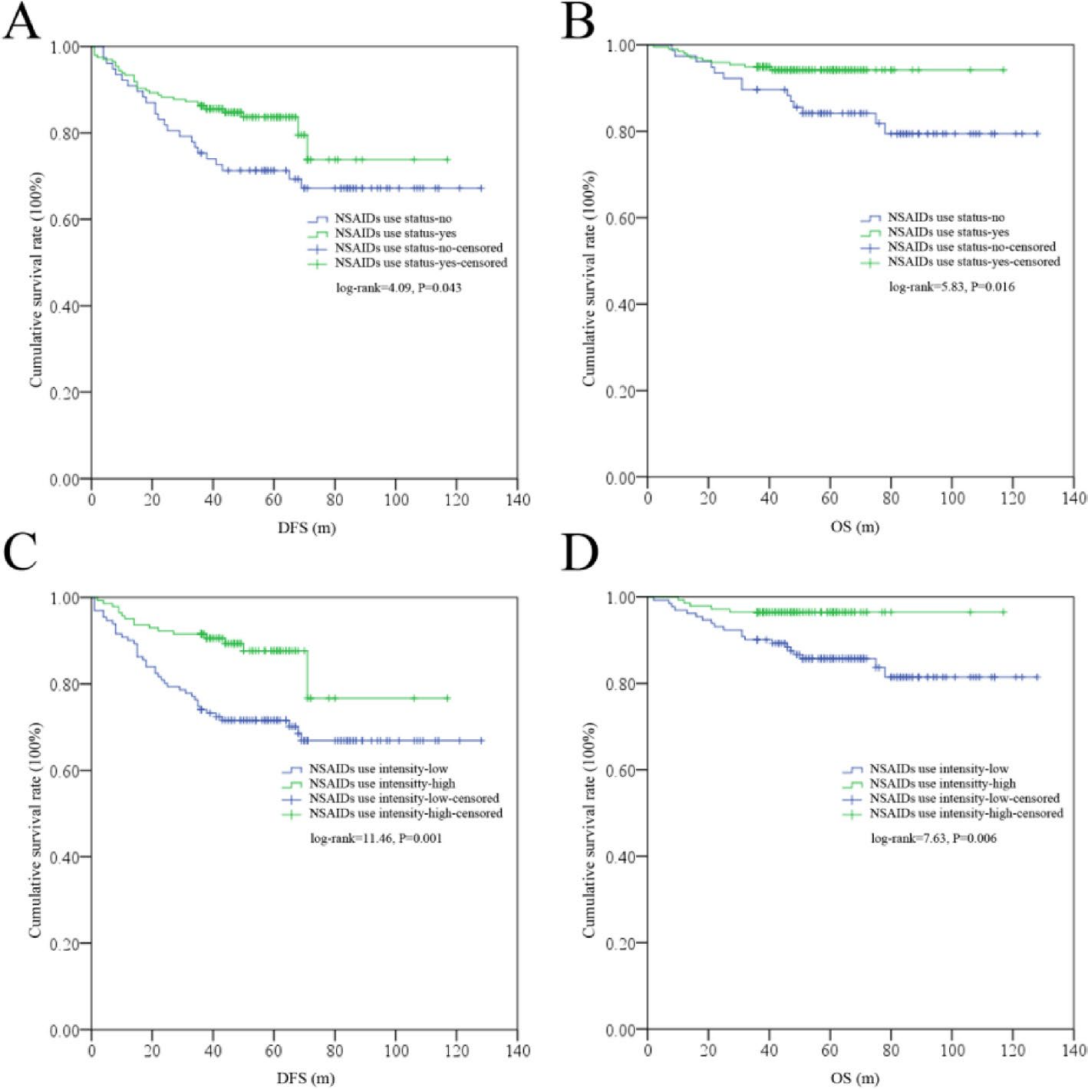
The survival differences among US no or yes and UI low or high subgroups. **A** DFS differences among US no or yes subgroups. **B** OS differences among US no or yes subgroups. **C** DFS differences among UI low or high subgroups. **D** OS differences among UI low or high subgroup. US, use status; UI, use intensity; DFS, disease-free survival; OS, overall survival

By Kaplan–Meier tests, significant differences for DFS (log rank = 11.46, *P* = 0.001) and OS (log rank = 7.63, *P* = 0.006) were found in UI low or high subgroups; similarly, significant difference was also found for DFS (log rank = 4.09, *P* = 0.043) and OS (log rank = 5.83, *P* = 0.016) in US no or yes subgroups (Fig. 3).

### Univariate and multivariate tests to validate the risk factors for survival

By using the Cox hazard model, it was found that age, sex, pathology, smoking status, N stages, TNM stages, and UI were shared risk factors both for DFS and OS, whereas T stages were identified as additional risk factors for DFS and alcohol status were identified as an additional risk factor for OS (Table 2). Subsequently, these factors were entered into the multivariate tests for DFS and OS, respectively; the results indicated that UI was one of the independent factors for DFS (*HR*: 0.50, 95% *CI*: 0.29–0.87, *P* = 0.014) but not OS (Table 3).

**Table 2:** Determination for risk factors for DFS or OS by univariate tests

	**DFS**		**OS**	
	***P***	**HR (95% *CI*)**	***P***	**HR (95% *CI*)**
**Age (y)**				
< 60	1		1	
≥ 60	0.033*	1.78 (1.05–3.01)	0.072	2.09 (0.94–4.64)
**Sex**				
Male	1		1	
Female	0.001*	0.37 (0.21–0.66)	0.002*	0.19 (0.06–0.55)
**Type of resection**				
Lobectomy	1		1	
Segmentectomy	0.105	1.86 (0.88–3.95)	0.100	3.37 (0.79–14.32)
**Pathology**				
ADC	1		1	
SCC	0.196	1.84 (0.73–4.67)	0.023*	3.52 (1.19–10.44)
Others	0.006*	4.28 (1.53–12.02)	0.091	3.54 (0.82–15.34)
**Smoking status**				
Never	1		1	
Current + former	< 0.001*	3.19 (1.88–5.40)	< 0.001*	4.45 (1.96–10.08)
**Alcohol status**				
Never	1		1	
Current + former	0.126	1.51 (0.89–2.57)	0.024*	2.49 (1.13–5.49)
**Comorbidity**				
With	1		1	
Without	0.880	0.95 (0.52–1.75)	0.295	0.57 (0.19–1.65)
**T stages**				
T_1_ + T_2_	1		1	
T_3_ + T_4_	0.002*	5.16 (1.86–14.31)	0.058	4.07 (0.95–17.37)
**N stages**				
N_0_	1		1	
N_1_ + N_2_	< 0.001*	9.70 (5.63–16.71)	< 0.001*	14.47 (6.26–33.46)
**TNM stages**				
I	1		1	
II	< 0.001*	7.06 (3.66–13.64)	< 0.001*	9.34 (3.50–24.92)
III	< 0.001*	20.36 (12.74–50.49)	< 0.001*	24.23 (9.23–63.60)
**NSAIDs UI**				
Low	1		1	
High	0.001*	0.38 (0.21–0.68)	0.010*	0.27 (0.10–0.73)

*UI* use intensity, *ADC* adenocarcinoma, *SCC* squamous carcinoma, *DFS* disease-free survival, *OS* overall survival

^*^With significant difference

**Table 3:** Determination for risk factors for DFS or OS by multivariate tests

	**DFS**		**OS**	
	***P***	**HR (95% *CI*)**	***P***	**HR (95% *CI*)**
**Smoking status**				
Never	1			
Current + former	0.007*	2.10 (1.22–3.62)		
**Alcohol status**				
Never			1	
Current + former			0.014*	2.75 (1.23–6.16)
**TNM stages**				
I	1		1	
II	< 0.001*	4.72 (2.35–9.50)	< 0.001*	8.90 (3.36–23.59)
III	< 0.001*	19.18 (9.49–38.78)	< 0.001*	26.78 (10.13–70.83)
**NSAIDs UI**				
Low	1			
High	0.034*	0.52 (0.28–0.95)		

*UI* use intensity, *DFS* disease-free survival, *OS* overall survival

^*^With significant difference

## Discussion

In this study, the use of NSAIDs during radical resection was considered to be correlated with the outcome in NSCLC patients. UI was found to be the most robust prognostic indicator for the use of NSAIDs in these patients, and UI high patients would have the superior outcome in contrast to the low ones. Moreover, UI was recognized as an independent risk factor for DFS.

Previously, a great number of epidemiological investigations have indicated the use of NSAIDs like aspirin and ibuprofen can reduce the incidence of cancers including lung cancer [21–24]. As in cancer patients, these agents also maintained a positive role in the patients’ outcome. For example, Giampieri et al. in a study with 66 previously heavily treated metastatic colorectal cancer found that aspirin could obviously improve the survival [25]. With regard to lung cancer, Chuang et al. in a large retrospective study with 38,842 inoperable NSCLC patients found the use of aspirin correlated with improved OS [26]; Kanda et al. in a study with 217 stages IIIB and IV NSCLC patients also found the use of loxoprofen sodium that could extend the survival in older NSCLC patients [27]. In recent years, with the popular target and immunotherapies in NSCLC, the value of the use of NSAIDs was also explored with these therapies. For example, Liu et al. in a study with 365 metastatic EGFR-mutant NSCLC patients received osimertinib ± aspirin and found that a combination of aspirin with osimertinib could improve the progression-free survival (PFS) and OS [15]; similarly, Aiad et al. in a study with 500 stages I–IV patients received immunotherapies ± aspirin and also found that the combination of aspirin could extend the outcome [16]. However, it was also notable that some studies suggested that use of celecoxib could have less positive role in combination with gefitinib [17], docetaxel, or other platinum-based chemotherapy [18, 28]. Nonetheless, all these studies were conducted in advance or metastatic staged settings. Up to date, only one study has explored the use of the NSAIDs in postoperative stages I–III NSCLC patients, and the results indicated the use of the NSAIDs correlated with good PFS and OS [19]. In addition, the use or not (corresponding to the US in our study) was found to be an independent risk factor both for PFS and OS [19]. However, this study only includes the use of indomethacin and ibuprofen which aimed to treat the postoperative fever; other types of NSAIDs or other intentions of the use of these agents were not involved. Indeed, some previous studies in colorectal cancer and breast cancer have validated the protective role of aspirin in adjuvant settings [29, 30]. Our study for the first time supported the protective role of the use of NSAIDs in addition to indomethacin and ibuprofen in NSCLC patients in postoperative scenario, which was partly in line with previous studies in adjuvant settings [19, 29, 30]. In addition, none of the patients received celecoxib, which is also in line with previous results with these agents in aforementioned advanced or metastatic cases.

It continued to be lacking of well-acknowledged definition concerning the use and use intensity of NSAIDs in cancer patients particularly in postoperative background. Previously, some studies have defined the aspirin or other NSAIDs exposure in cancer patients. For example, Liao et al. defined the use of aspirin as one or more prescriptions recorded before and after the diagnosis with any dosage [31], whereas in Giampieri et al.’s study, the definition of exposure was the ones taken for at least 12 months at a dose of at least 100 mg/day [25]; other studies defined various criteria of exposure of these agents [30, 32]. As in lung cancer, Chuang et al. defined the aspirin users as those who used it for > 28 defined daily doses after diagnosis [26]; Jiang et al. defined the use or not of NSAIDs (only indomethacin and ibuprofen) as the standardized dosage in clinic [19], whereas in reports about the combination of it with osimertinib or immunotherapies, the definition of exposure was obscure [15, 16]. Interestingly, all these studies are conducted retrospectively. However, in prospective clinical trials about celecoxib, its exposure was rigorously defined with fixed dosage concurrent with gefitinib [17] or chemotherapy [18]. In our study, all the patients received daily dose of the NSAIDs irrespective of the categories, and we thus refer to Jiang et al.’s and Liao et al.’s study [19, 31] to define the exposure as US; in addition, we also explored the prognostic value of AD and UI in these patients. The results indicated UI displayed the highest AUC among these indexes and was found to be an independent risk factor for survival. Our results for the first time indicated that the UI may a reasonable index for the use of NSAIDs in these patients; however, more studies are still required to validate our speculation in future.

Mechanically, the role of NSAIDs in regulating lung cancer cells has been under extensive study. For example, aspirin could manipulate the miR-98/WNT1 axis to inhibit cancer progression [33]; it could also suppress the growth of cancer cells via targeting the TAZ/PD-L1 axis [34]; in addition, other NSAIDs like ibuprofen could enhance the effect of cisplatin by suppressing the heat shock protein 70 [13], and acetaminophen could promote ferroptosis by regulating Nrf2/heme oxygenase-1 signaling pathway [35], and loxoprofen sodium could inhibit tumor growth by suppressing vascular endothelial growth factor in a mouse model [36]. In recent years, the key role of circulating tumor cells (CTCs) has been identified in tumor recurrence and metastasis [37, 38]; in particular, some of these cells presented features that are similar to the so-called cancer stem cells (CSCs) in many cancers [39, 40] including lung cancer [41, 42], the latter of which are characterized by high potential of self-renew and multiple treatment resistance, and complete removal of these cells was regarded as the ultimate approach to cure the disease [43–45]. In lung cancer, it was notable that the CTCs could be found in up to 51.8% stages I–III radically resected patients before surgery [38], and these cells could be even found in 96.5% patients during surgery in pulmonary vein [46]. Notably, the use of some NSAIDs like aspirin could decrease the CTCs numbers in colorectal and breast cancer patients [47]; moreover, aspirin could have a broad spectrum of inhibition for many CSCs (including lung cancer) by complex mechanisms [48, 49]. Based on these facts, it was notable that the use of NSAIDs after surgery could potentially reduce the quantity of the CTCs and in particular eliminate a cluster of these cells featured like CSCs, which could then improve the patients’ outcome. Except these, it was well established that inflammation plays an important role in cancer initiation, recurrence, and metastasis [50]. Some cytokines, like IL-6, could be remarkably elevated in lung cancer patients [51] in particular for those who underwent surgery [52]. It is noteworthy that IL-6 could not only promote the cell proliferation [53], induce treatment resistance [54, 55], and promote metastasis [56] in lung cancer but also promote the expansion of the CSCs [57]. Interestingly, some NSAIDs like aspirin could not only cancel the pro-tumorigenic effects of IL-6 [58] but also downregulate the IL-6-STAT3 signaling pathway to induce cancer cell apoptosis, which have been validated in colorectal cancer [59] and glioblastoma A172 cells [60]. Based on these facts, it was also plausible that the use of NSAIDs may also have a role in interrupting the correlation of inflammation and cancer in NSCLC, which may result in a sound outcome for these patients.

Our study could have some clinic implications. First, taking into consideration the evolution of lung cancer cells during its development [61] and the role of NSAIDs in NSCLC, it would be reasonable to take these agents routinely as a tertiary chemoprevention of the disease immediately after surgery; second, since low-dose aspirin (< 75 mg/day) rarely contribute to improve cancer-specific mortality in lung cancer [62] and could even promote the cell growth [63], it was recommend that these patients should take the NSAIDs with a relatively high dose (like *UI* ≥ 74.55 mg/day in our study); however, adverse effects like gastric erosions and ulcers should also be balanced in clinic [64]. There are also some limitations for present study. First, it was a retrospective study with limited sample, the cases in US no groups were only 77, and potential bias cannot be satisfactorily ruled out; second, the pharmacokinetics and pharmacodynamics of different NSAIDs were not identical, we still lack strong evidence to support the convert ratio as 1 for each other in our study, and there are still lack of relevant studies to full support our explanations for the role of these agents in NSCLC except aspirin; and third, although UI was found to be a robust prognostic index compared to US and AD, the question for the duration of these agents cannot be answered at present. We advocated more studies; in particular, those prospective clinical trials should be carried out to validate our results in future.

## Conclusion

As a summary, we found that the use of NSAIDs during radical resection in NSCLC patients correlated with the outcome, and patients with a relative high UI have better outcome.

## Data Availability

The datasets generated or analyzed during the current study are available from the corresponding author (Minbiao Chen) on reasonable request.
